# Performance of medical students on a virtual reality simulator for knee arthroscopy: an analysis of learning curves and predictors of performance

**DOI:** 10.1186/s12893-016-0129-2

**Published:** 2016-03-25

**Authors:** Stefan Rahm, Karl Wieser, Ilhui Wicki, Livia Holenstein, Sandro F. Fucentese, Christian Gerber

**Affiliations:** Orthopaedic Department, Balgrist University Hospital, University of Zurich, Forchstrasse 340, CH-8008 Zurich, Switzerland

**Keywords:** Education, Learning curve, Predictive factors, Virtual reality, Knee arthroscopy

## Abstract

**Background:**

Ethical concerns for surgical training on patients, limited working hours with fewer cases per trainee and the potential to better select talented persons for arthroscopic surgery raise the interest in simulator training for arthroscopic surgery. It was the purpose of this study to analyze learning curves of novices using a knee arthroscopy simulator and to correlate their performance with potentially predictive factors.

**Methods:**

Twenty medical students completed visuospatial tests and were then subjected to a simulator training program of eight 30 min sessions. Their test results were quantitatively correlated with their simulator performance at initiation, during and at the end of the program.

**Results:**

The mean arthroscopic performance score (z-score in points) at the eight test sessions were 1. -35 (range, -126 to -5) points, 2. -16 (range, -30 to -2), 3. -11 (range, -35 to 4), 4. -3 (range, -16 to 5), 5. -2 (range, -28 to 7), 6. 1 (range, -18 to 8), 7. 2 (range, -9 to 8), 8. 2 (range, -4 to 7). Scores improved significantly from sessions 1 to 2 (*p* = 0.001), 2 to 3 (*p* = 0.052) and 3 to 4 (*p* = 0.001) but not thereafter. None of the investigated parameters predicted performance or development of arthroscopic performance.

**Conclusion:**

Novices improve significantly within four 30 min test virtual arthroscopy knee simulator training but not thereafter within the setting studied. No factors, predicting talent or speed and magnitude of improvement of skills could be identified.

## Background

Simulator training is gaining popularity and there are many reasons for the rising interest for its role in orthopaedic resident selection and education. The restriction of work hours of residents directly correlates with fewer hours in the operating room observing and performing operations under supervision [1], to the extent that residents do not feel prepared for this technically difficult field after regular residency training [2]. For nearly every joint, arthroscopy simulators have been built and it has been shown that training on simulators improves simulator skills but a clear correlation between simulator training and improved arthroscopic skills in the operating room has not yet been fully confirmed [3–6]. Only in one study until this time the transfer validity has been shown [7]. The role of factors potentially facilitating surgical skill development such as parental job, experience with computer games or talents like spatial sense or manual skill or others have not been assessed.

For the selection of a future arthroscopic surgeon it would be helpful to know whether factors outside the operating room, respectively even outside the arthroscopic simulation could identify a certain potential for becoming a skilled arthroscopic surgeon. This fact could be path breaking to select a specialty for both, the educator and the candidate. In non-orthopaedic surgical fields there have been studies testing the aptitude of future surgeons with psychomotor testing. It seems to be useful but yet debatable even though baseline visuospatial abilities have been shown to correlate with operative skill [8–14].

So far it is not known whether visuospatial and/or other factors are predictive for arthroscopic simulator performance. We therefore designed a prospective study to analyze the learning curve and test the hypothesis that baseline factors and measures of visuospatial, motor and mathematical abilities of medical students could predict knee simulator performance and the progression of it using a validated reality-based knee arthroscopy simulator.

## Methods

### Participants

Prior participation of the study, all participants gave their oral and written informed consent. The IRB waived the need for ethical approval. (Kanton Zürich, Kantonale Ethikkommission: Nr: 10-2016) Twenty medical students (11 females and 9 males; 5 left handed and 15 right handed) recruited from the University of Zurich with a mean age of 23 years (range 19 to 34) between the first and the fifth year of medical school volunteered to participate in this study.

#### Arthroscopic simulator course protocol

The system consists of a passive haptic knee arthroscopy simulator with a right plastic knee mock up and given lateral and medial portals. During all the exercises the participants were accompanied and supervised by one of the co-authors. All participants received an identical standardized instruction to the simulator and were taught how to manage the 30 degree arthroscope and the tools (standard punch and shaver to perform partial meniscectomy), which had to be used during the exercises. Further, all the participants could get familiar with the simulator for exactly 3 min using the camera through the lateral portal and instruments through the medial portal. The knee simulator used in this study has been previously described and its face and construct validity has been established and reported [15].

In week 0 – 4 all participants passed a standardized simulator training including eight sessions with six exercises (30 min) during 4 weeks.

There were three identical exercises which had to be done during all eight sessions to get a continuous record of all the participants. The first exercise was to test the triangulation, the second exercise to test the handling of a partial meniscectomy with the punch and/or the shaver and the third exercise to test the removal of foreign bodies (stars). Additionally, at each session three new tasks of increasing difficulty were introduced to evaluate the learning curve when adapting to a new situation. These three new tasks again consisting of triangulation, partial meniscectomy and removal of foreign bodies in different variations. There was a time limit of five minutes in all exercises.

After performing all the above exercises the metrics from the simulator (operation time in seconds and overall distance in centimetres of the tools and camera inside the knee simulator during a task) were extracted from the computer. Furthermore the number of foreign bodies which had been removed were counted.

Each practice session was completed except for participant 12 who did not finish his last practice session. Therefore score 8 of participant 12 is missing.

### Design

**Fig. 1:**
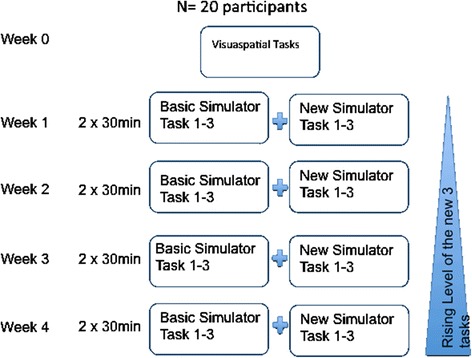
Principals of method. In this figure the precise study set up and the course of action is visualized

In Fig. 1 the study principals of our methods are depicted. In week 0 all participants fulfilled a questionnaire and underwent tests to measure the manual skill, spatial sense and mathematical skills.

### Demographic data and questionnaire

The questionnaire consisted of different questions regarding demographic data (age, years in medical school, gender, dexterity), sports activities, playing an instrument, playing videogames, having operating room experience, parents (medical) profession and their planned future medical (surgical) specialty (Table 1).

**Table 1:**
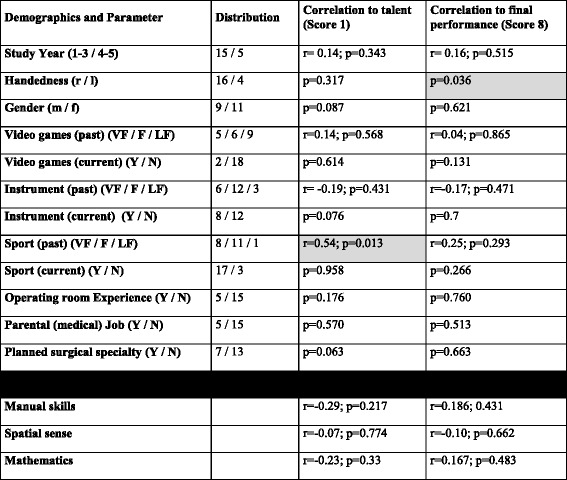
In this table the relevant demographic and information of the questionnaire with the associated correlation to the first (score 1) and the final (score 8) performance is depicted. The statistically significant correlations are gray highlighted and include the positive correlation of playing frequently sport to the first performance (score 1). Secondly, in the final score 8 all the participants were right handed and the left handed participants had a low final score 8 why this was significant

In the binary results the Mann–Whitney-*U* test was performed and *p*-value was given. In the other continuous and ordinal variables the Spearman rank correlation test was performed and r- and the *p*-value depicted

*Abbreviations*: *r* right, *l* left, *m* male, *f* female, *VF* very frequent, *F* frequent, *LF* less/not frequent, *Y* Yes, *N* No

### Test A: Manual skill

The ESCAPA manual skill test (Reference: http://cisa85.de/tmp/MultitaskingTest.htm) is used in the US-Airforce in the evaluation of pilots. It tests a fine motor manual computer skill with the practiced extremity using a computer mouse.

ESCAPA is played in a small white box, in which there are five rectangles: four move automatically; and a square can be moved by the player. The object of the game is to navigate the square by dragging it with the mouse within the small white playing field for as long as possible, avoiding both the randomly moving blocks, and the surrounding border. The time was taken in seconds for this skill.

### Test B: Spatial sense

This included a test, which is used in the accept ance test of all swiss medical schools (Reference: http://www.ztd.ch/w/index.php?title=EMS#Berufseignung_und_Studieneignung).

**Fig. 2:**
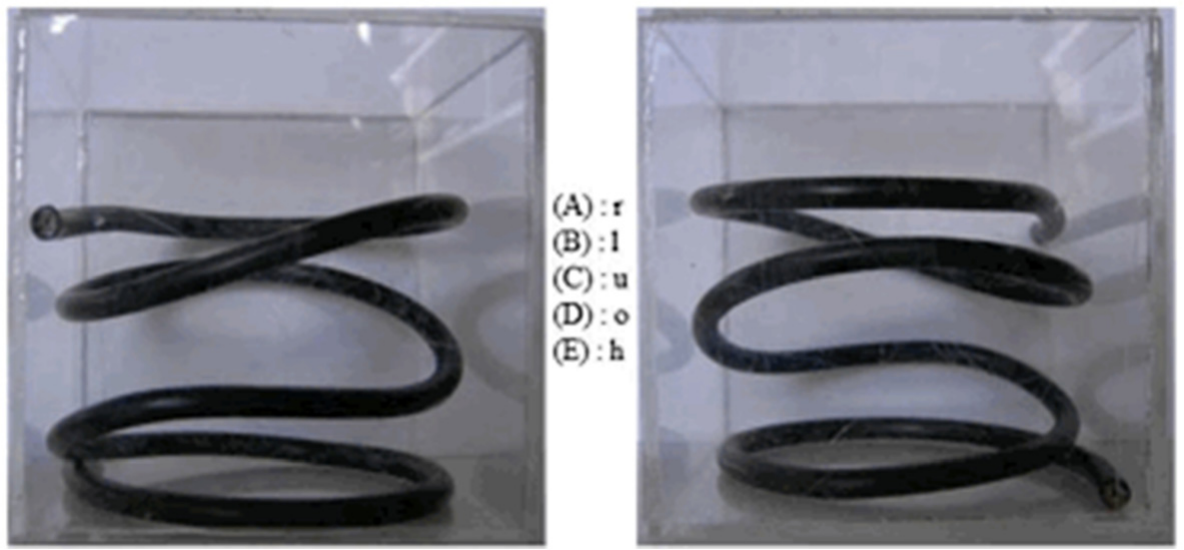
Example of a mental rotation skill: hose figure. Here is an example of a hose figure where the spatial sense is tested. The participant was given 24 such figures and they were able to choose an answer from A to E. Only one answer was correct. The left picture shows the original view (front view) and the right pictures shows the rotated cube. A: view from the right side, B: view from the left side, C: view from below, D: top view, E: back view. In this example E is the correct answer

It tests the spatial sense and mental rotation and was proven to correlate with passing the complete medical school exams achieving the Swiss medical degree according to the Swiss medical board. The test included five different hose figures, which were three-dimensional arranged, which had to be rotated mentally in a correct way. 24 of those exercises had to be completed in 12 min (Fig. 2).

### Test C: Mathematics

**Fig. 3:**
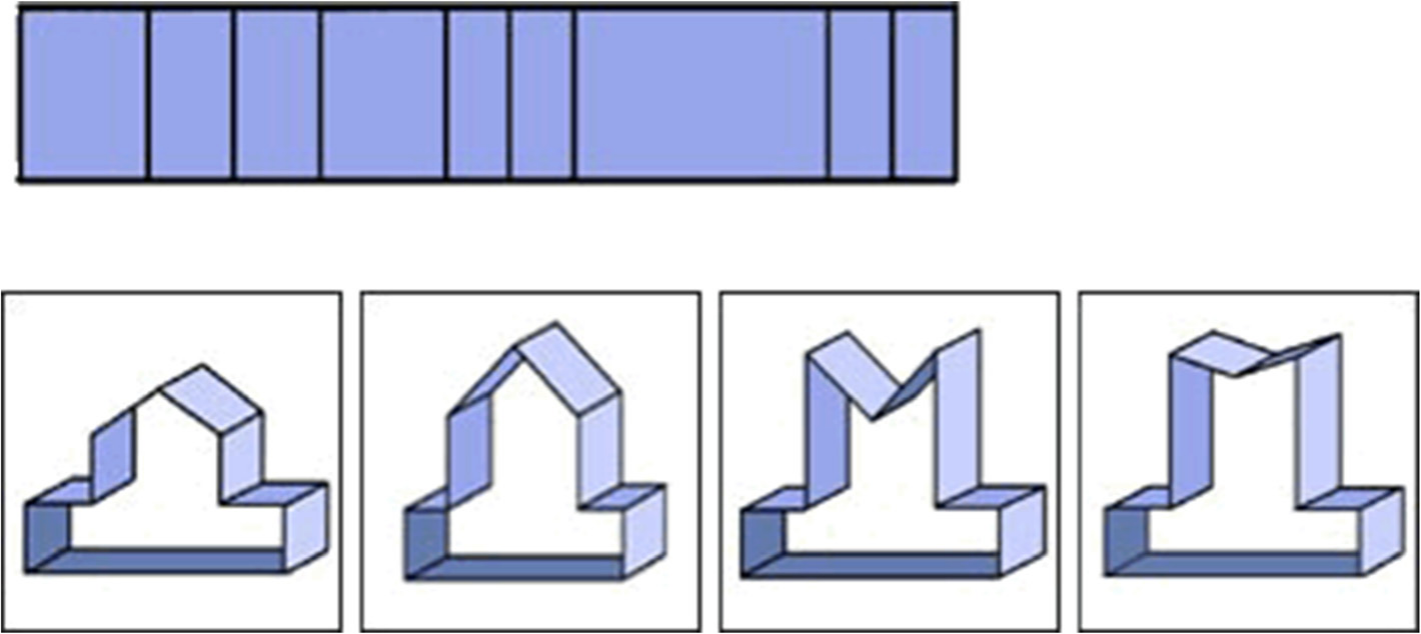
Example of a mental construction skill: the relationship between plane shapes and solid figures. In this task another aspect of spatial sense is tested, i.e. mental construction skill. The participant was given 12 such figures and they were able to choose an answer from A to D. Only one answer was correct. In this example C is the correct answer

In this test, a two-dimensional representation of a three-dimensional object is given. The task is to figure out which of the 4 three-dimensional objects is the correct representation of the given two-dimensional map. Twelve of those exercises had to be completed in 10 min (Fig. 3). This exercise is used in IQ tests and gives an impression of the spatial sense and mental construction skill (Reference: http://www.fibonicci.com/de/raumliches-vorstellungsvermogen/test-schwierig/).

### Statistical analysis

The statistical analysis and the development of the z-score was performed by an independent professional biostatistician using the SPSS version 20 for Mac (SPSS, Inc., Chicago, IL). To compare the performance of the students, we used a standard score (z-score) built out of the means from the sessions 4 to 8 of all participants concerning time, time per task (item), camera and instrument distance in centimeters and the number of removed foreign bodies. In the first exercise “triangulation”, we used the camera path length, the hook path length and time per item (= subscore 1-3). In the second exercise “partial meniscectomy”, we used camera path length, total path length for all the instruments and total time (= subscore 4-6). In the third exercise “removal of foreign bodies”, we used the camera path length, the punch path length and time per item (= subscore 7-9). Therefore, every student has had one z- score built out of 9 subscores for each of the eight sessions of the exercises. Every pre-test parameter and all demographic factors were correlated to the first and the last test performance. Furthermore, each of the eight z-scores were correlated to the final test result to analyse if the final performance could be predicted to an earlier stage.

The z-scores were correlated with continuous and ordinal variables using the Spearman rank correlation test. Groups were compared with the Mann–Whitney *U* test. Scores at different points of time were compared using Wilcoxon’s signed ranks test. A *p*-value < 0.05 is considered as significant.

## Results

### Demographics

The relevant data and the distribution of the questionnaires so as the correlation of it to the first score 1 and final score 8 are summarized in Table 1.

### Scores

**Fig. 4:**
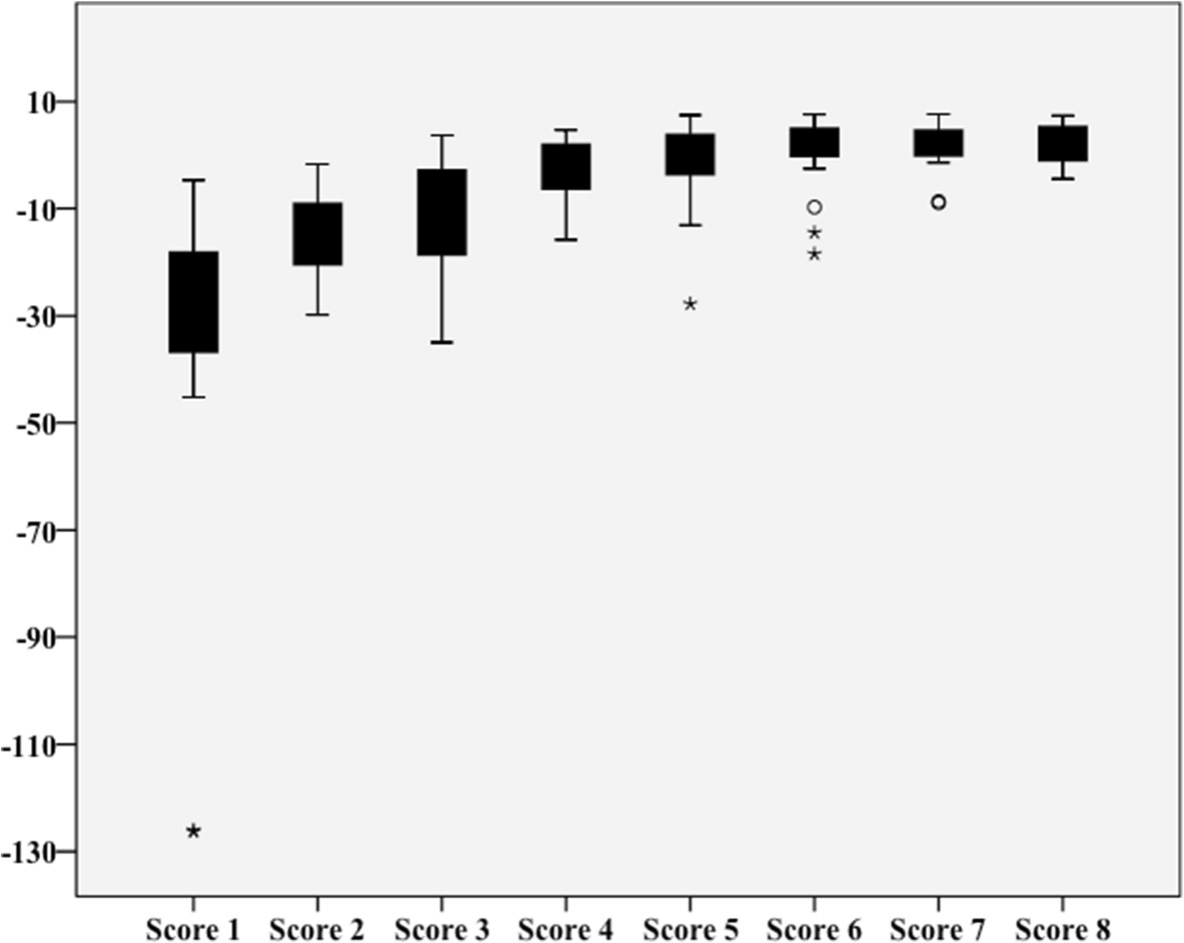
Boxplots of the 8 z-scores. In this boxplot the results of the z-scores are depicted. It is seen that there is a relevant development of the z-score throughout the whole training which flattens after the forth exercise. The participants ended with high scores in the seventh and eighth exercise with also relevantly less distribution of the standard error

Analyzing the learning curve of the participants, we found a relevant improvement during the first four exercise sessions (score 1–2 (*p* = 0.001), score 2–3 (*p* = 0.052), score 3–4 (*p* = 0.001). Thereafter the learning curve reached a plateau with only slight mean improvement of performance (score 4 – 5, *p* = 0.332; score 5 – 6, *p* = 0.057; score 6 – 7, *p* = 0.681; score 7 – 8, *p* = 0.445). Further we found decreasing variability of the participants’ performance, indicated by gradually decreasing standard deviations from 33.2 points in score 1 to 3.8 points in score 8 (Fig. 4). In the appendix the Table 2 shows the complete scores of all the eight tests and 20 patients.

**Table 2:** This table shows the summary of the z-score results in all eight exercises. Fig. 4 is depicting the results in a boxplot format

Participant	Score 1	Score 2	Score 3	Score 4	Score 5	Score 6	Score 7	Score 8
P 1	-16.6	-16.7	-25.1	-15.9	-0.6	-0.6	0.4	-3.9
P 2	-24.1	-15.0	-5.8	1.3	3.6	2.8	1.0	1.5
P 3	-31.9	-9.2	-15.4	-2.4	5.3	2.3	-1.4	3.9
P 4	-34.3	-9.4	3.2	-0.9	-0.1	3.3	4.5	3.3
P 5	-9.9	-10.4	-3.3	2.6	3.6	3.0	4.1	5.3
P 6	-38.7	-13.7	-16.3	-6.0	-10.1	5.2	6.0	0.9
P 7	-20.4	-8.3	-18.6	-6.4	1.8	1.6	3.5	-0.6
P 8	-126.3	-27.6	-19.4	-10.6	-27.7	-14.5	-1.2	3.2
P 9	-20.9	-28.7	-18.2	-15.1	-4.6	-18.5	-9.0	-1.5
P 10	-21.2	-29.8	-35.0	-6.1	-13.1	-9.7	-8.7	-4.4
P 11	-4.7	-1.7	3.7	4.7	6.0	7.2	4.5	4.5
P 12	-43.2	-17.9	-3.0	-5.5	-0.6	0.8	-0.1	
P 13	-27.0	-25.1	-12.1	-0.4	-7.8	4.8	3.2	3.6
P 14	-32.5	-17.3	-7.4	1.6	3.9	4.7	2.2	7.0
P 15	-20.3	-19.6	1.4	-0.7	-2.1	1.6	0.4	-0.9
P 16	-11.8	-3.1	1.8	3.5	2.6	2.5	3.9	4.9
P 17	-45.2	-20.9	-14.7	-5.4	-1.8	-2.6	2.9	-3.8
P 18	-10.0	-7.9	-3.1	1.8	0.3	4.7	4.3	6.1
P 19	-32.4	-17.5	-3.2	3.3	7.5	7.6	7.6	5.2
P 20	-126.1	-12.4	-27.1	1.2	0.2	5.5	4.5	7.4
Median	-25.5	-15.9	-9.8	-0.8	0.0	2.6	3.1	3.3
Mean	-34.9	-15.6	-10.9	-2.8	-1.7	0.6	1.6	2.2
SD	33.2	8.1	11.0	6.0	8.1	7.0	4.3	3.8
Max	-4.7	-1.7	3.7	4.7	7.5	7.6	7.6	7.4
Min	-126.3	-29.8	-35.0	-15.9	-27.7	-18.5	-9.0	-4.4

*P* participant

### Correlation

Only right handedness correlated substantially to the final simulator perfomance (score 8) (*p* = 0.036). Being active in sports in the past was a very weak but still significant predictor of better performance at the very first session (score 1) (*r* = 0.54; p: 0.013). No other examined pretest or demographic parameter showed a correlation with the final simulator performance (Table 1).

Whereas there was a significant correlation of score 4 to the final performance (score 8) *r* = 0.71; *p* < 0.0001 there was no such correlation of score 1 to the final performance (score 8) *r* = 0.02; *p* = 0.925.

## Discussion

This study shows that medical students, participating in a standardized virtual-reality based knee arthroscopy simulator training program, have a significant, but surprisingly steep and short learning curve. This improvement of skills was relevant until the forth test (score 4), which represents about two hours of training, without significant further improvement afterwards. Reasons for this plateau are hypothetical, but this learning curve is consistent with other results in the current literature [3, 16, 17] and might be a result of the so far limited possibility to simulate difficult arthroscopically guided surgical interventions (i.e. meniscal repair, ACL reconstruction). Since the first score does not correlate with the last score, it would be unreasonable to grade a medical student after the first 30 min of arthroscopy of simulator training and it is notable that the the participant who ultimately reached the highest performance in the final session, was amongst the five weakest participants in the first session. This is in sharp contrast to the correlation of score 4 to the final score 8. When looking at the five best participants in score 4 they reached the very high ranking of 3, 4, 5, 6 and 7 in the final score 8. Further the weakest five participants in score 4 reached the low ranking of 11, 15, 17, 19 and 20 in the final score 8. These results show that after a period of two hours of training a reasonably precise predictive statement can be made regarding the future performance of a participant. How a two hour assessment could be carried out during an application process might deserve further study.

Disappointingly, we were unable to identify strong and relevant factors identifying “talented” arthroscopic surgeons. The only factor which correlated significantly to the final performance was to be right handed. This fact might be biased by the fact that an anterolateral portal of a right knee was used for the arthroscope which therefore was held by the left hand. The arthroscopic working tools were inserted through the anteromedial portal and were therefore used with the right hand. In the tested simulators current version, there is only a right knee available even though it should bi possible to change it in the future. The only other factor, which showed a significant but weak correlation to the primary test result was the sportsmanship in the past, but it did afterall not predict final simulator performance. Whether this test result could be related to athletically active persons are more competitive and try harder at the first effort is unknown but a possibility.

Our findings were in contrast to previous laparoscopic simulator studies, which were able to identify different predictive factors. Risucci et al. [13] suggested that age, experience and visual spatial perceptual ability may play a role in determining the speed a surgeon can acquire and perform laparoscopic skills using a laparoscopic intracorporal simulator. Madan et al. [18] found that the only predicting factor, which correlated with the laparoscopic simulator performance was eating with chop sticks. There were, however no visuospatial or other tests used in the study of Madan et al. as predicting factors. Stefanidis et al. [12] showed that residents training on a laparoscopic simulator performed 50 % better after a mean of 12 h and that the only test, which correlated with the simulator performance was the visuospatial test “card rotation”, which is similar to the visuospatial test used in this present study. Further Stefanidis et al. found training duration and repetition correlated with prior video gaming, billiard exposure, grooved pegboard (time to place different pegs with a key with one hand in a pegboard), finger tapping with the index finger as fast as possible and map planning (a mental test for visuospatial capability and concentration).

We are aware of the potential limitations of this study. Although this study was prospectively conducted, the limited number of participants (*n* = 20) was possibly too small to detect subtle differences but did allow to exclude substantial differences and trends in the study group which would have made impact on selection of trainees. Furthermore, we are aware of the fact that in arthroscopic simulator surgery there have been attempts to not only measure performance with metric parameters but also with subjective scores. We however considered the Arthroscopic Surgery Skill Evaluation Tool (ASSET) [19], which seems to be a valid and reliable pass-fail examination of diagnostic knee arthroscopy simulation, as being not precise enough to quantitatively evaluate and compare the longitudinal participants performance. Furthermore as this was the first study investigating predictive factors in arthroscopic simulator training there were no established guidelines about visuospatial, manual or mathematical tests. Therefore it remains hypothetical if the chosen tests were either not appropriate to detect the expected predictive factors, or it is simply not possible to predict simulator performance using demographics or psycho-motoric tests.

Despite the above mentioned limitations, this study revealed some interesting information about potential arthroscopy simulator training programs and the potential use recruiting medical students in orthopaedic residents programs. Medical students without any previous arthroscopic experience improve significantly after a two-hour training virtually based simulator program of increasing difficulty levels. The level which is reached after this two hour training on the arthroscopy simulator does not change relevantly after training of more than 2 h. As transfer validity of simulator training in to the operating room was proven before [20], even though this was on an other virtual reality based arthroscopic simulator, it seems reasonable to state that these trainees do not only improve there simulator skills, but thereof should be able to benefit in real arthroscopic surgeries.

## Conclusion

There is a significant learning curve of medical students when completing a standardized training program performed on a validated virtual-reality-based knee arthroscopy simulator during the first 2 h of training. The “good” and the “less good” arthroscopic surgeon can then be evaluated and further training will not change that finding. We however failed to identify factors, which help to predict talent and potential development of arthroscopic skills. Further studies are needed to become more and precise information about the transfer validity to the operating room and the amount of training time and type to make a sophisticated recommendation about the potentially best educational program for future orthopaedic surgeons providing best health care for our patients.
